# *KDM2B* Is Implicated in Bovine Lethal Multi-Organic Developmental Dysplasia

**DOI:** 10.1371/journal.pone.0045634

**Published:** 2012-09-27

**Authors:** Stefania Testoni, Elena Bartolone, Marco Rossi, Andrea Patrignani, Rémy Bruggmann, Peter Lichtner, Jens Tetens, Arcangelo Gentile, Cord Drögemüller

**Affiliations:** 1 Department of Veterinary Clinical Sciences, University of Padua, Legnaro, Padua, Italy; 2 Veterinary Clinical Department, University of Bologna, Ozzano Emilia, Bologna, Italy; 3 Functional Genomics Center Zürich, University/ETH Zürich, Zürich, Switzerland; 4 Department of Biology, University of Bern, Bern, Switzerland; 5 Institute of Human Genetics, Helmholtz Zentrum München, German Research Center for Environmental Health, Neuherberg, Germany; 6 Institute for Animal Breeding and Husbandry, Christian-Albrechts-University Kiel, Kiel, Germany; 7 Institute of Genetics, Vetsuisse Faculty, University of Bern, Bern, Switzerland; University of Bonn, Germany

## Abstract

In the last decade breeders of Romagnola cattle observed an outbreak of a new congenital anomaly. This lethal multi-organ developmental dysplasia is mainly characterized by facial deformities, ascites and hepatic fibrosis. Affected stillborn calves were inbred to a single founder sire suggesting autosomal monogenic recessive inheritance. We localized the causative mutation to a 1.2 Mb interval on BTA 17 by genome-wide association and identical by descent mapping. A solution-based method for targeted DNA capture combined with massively parallel sequencing was used to analyze the entire critical region containing 24 genes. Homozygosity for two non-synonymous coding sequence variants affecting the *RNF34* and *KDM2B* genes was detected by evaluating one affected calf. Here we show that the disease phenotype is associated with a *KDM2B* missense mutation (c.2503G>A) leading to an amino acid exchange (p.D835N) in an evolutionary strongly conserved domain. In addition, the genetic makeup of three inbred cattle strongly supports the causality of the *KDM2B* mutation. This report of a naturally-occurring spontaneous mutation of a JmjC domain containing histone demethylase gene provides evidence for their important role in the endo- and mesodermal organ development.

## Introduction

**Figure 1:**
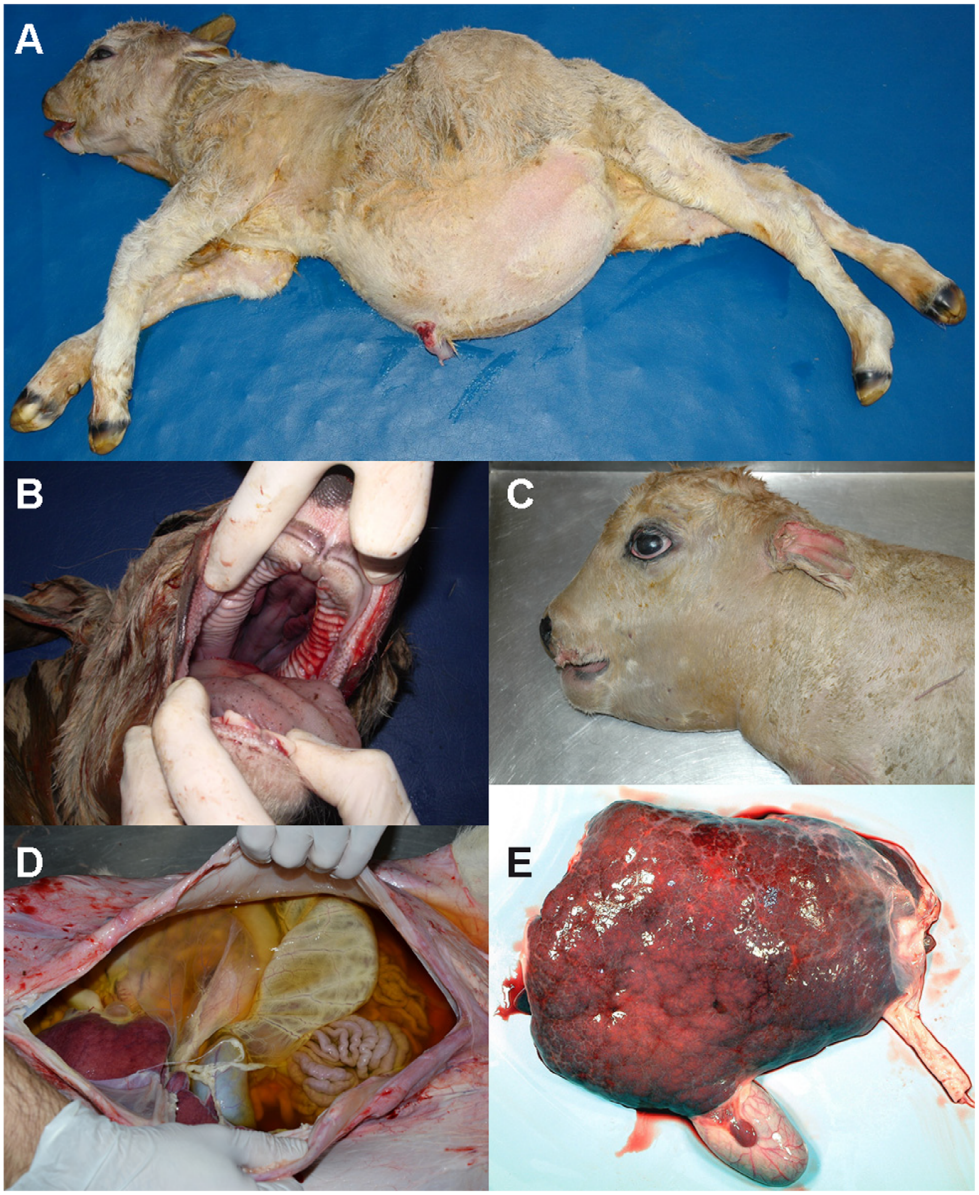
Phenotype of the paunch calf syndrome in Romagnola cattle. Note the shortened face and the abdominal distension with a considerable peritoneal liquid presence (A), and, in some cases, tongue protrusion and cleft palate (B), shortened and flattened splanchnocranium (C), accumulation of dark-yellowish, turbid fluid in the abdominal cavity (D), and irregular surface of the liver and presence of a hepatic cyst with reddish fluid content (E).

Since 2003 breeders of Romagnola cattle in Italy have experienced an increase in the occurrence of a previously unknown complex congenital anomaly, mainly characterized by craniofacial deformities, an enlarged fluid-filled abdomen and hepatic fibrosis [1]. Affected calves are usually stillborn and the disease is known as ‘paunch calf syndrome’ (Figure 1). The emerging paunch calf syndrome disease causes significant economic and animal welfare concerns. The paunch calf syndrome defect is supposed to be inherited as a monogenic autosomal recessive trait [1]. The outbreak of paunch calf syndrome can be attributed to selective breeding practices involving the extensive use of particular highly selected artificial insemination sires leading to an increase in co-ancestry and inbreeding [2]. Parents of affected calves do not present any clinical signs. Therefore, it is crucial that the breeders implement an effective strategy to control this recessive defect [1], [3]. Identification of the causative mutation has an immediate translation into breeding practice, allowing direct DNA testing and effective selection against the defect through avoidance of at-risk matings [2]. After sequencing the cow genome [4], [5] recent examples showed that the availability of genome-wide, high-density SNP arrays, combined with the typical structure of cattle populations, markedly accelerates the positional identification of genes and mutations that cause inherited recessive defects [2], [6], [7].

In humans and other mammalian species a comparable syndrome has also not yet been described. Lacking functional candidate genes for this unique phenotype, the spontaneous cattle paunch calf syndrome mutants provide the potential to gain insights into the pathogenesis of this developmental dysplasia affecting several endo- and mesodermal derived organs.

The aim of our investigation was to identify the mutation associated with paunch calf syndrome in Romagnola cattle using a genome-wide association study mapping approach, followed by targeted re-sequencing of the entire disease locus.

## Results

### The Paunch Calf Syndrome Mutation Maps to BTA 17

**Figure 2:**
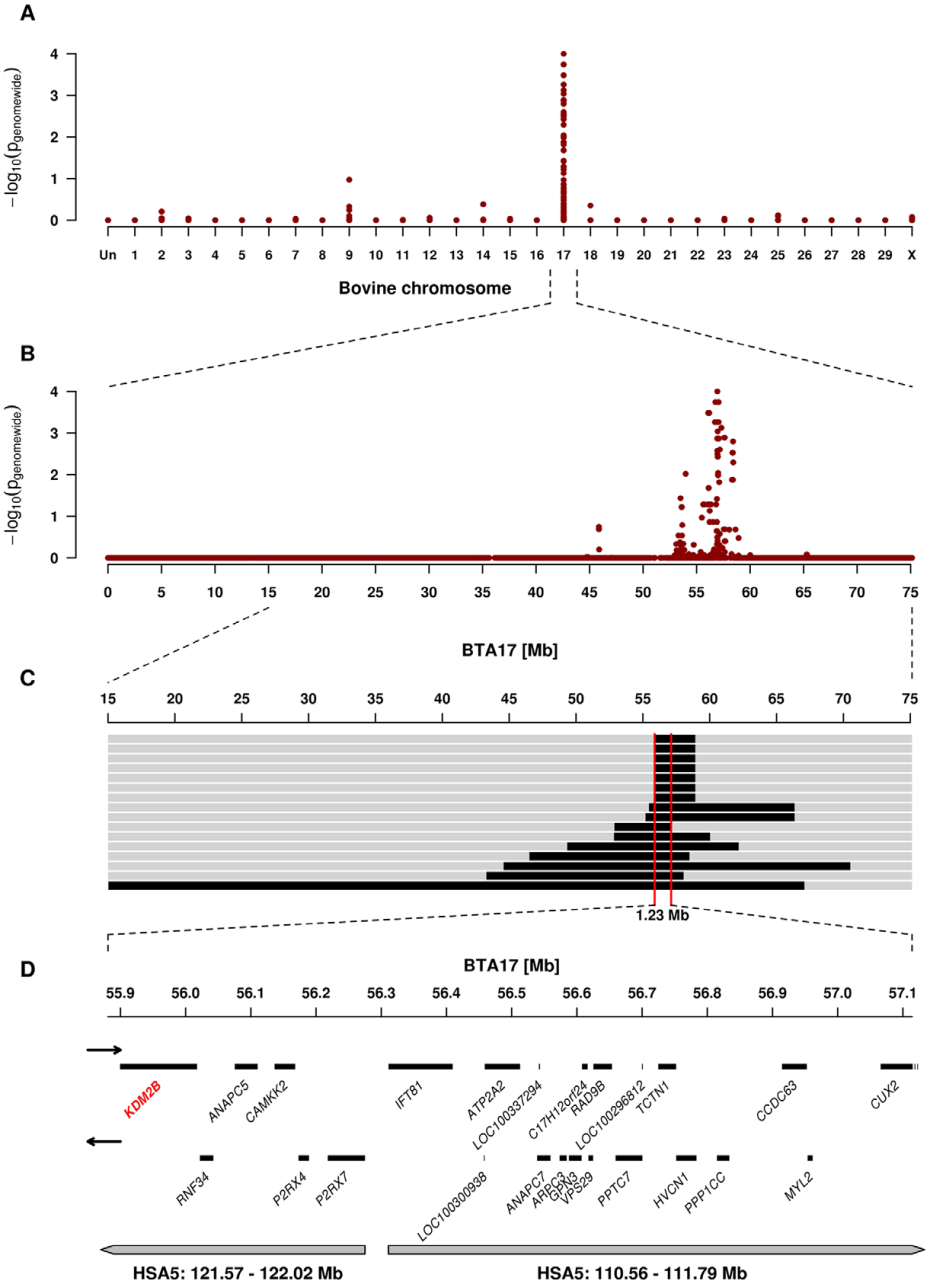
Genome-wide association and homozygosity mapping of the paunch calf syndrome locus. (**A**) Case-control genome-wide association analysis shows a significant association to SNPs on BTA 17. (**B**) Single SNP association results across BTA 17. (**C**) The limit of the homozygous 1.23 Mb interval shared by the 16 cases is highlighted in red. (**D**) Gene content of the corresponding human chromosome 5 segments. The *KDM2B* gene containing the causative mutation is highlighted in red.

We genotyped 777,962 SNPs in 16 paunch calf syndrome affected calves and 41 controls. After removing non-informative markers 536,171 SNPs were used for the subsequent analyses. A genome-wide association study revealed a single strong signal for paunch calf syndrome on bovine chromosome (BTA) 17 (Figure 2A). The best-associated SNP had a genome-wide corrected p-value of 1.0×10^−4^ at 56,938,011 bp on BTA 17 (Figure 2B).

The quite uniform phenotype and a high degree of inbreeding suggested the existence of a single recessive founder mutation. Pedigree analysis indicated that all paunch calf syndrome cases with available pedigree records traced back to a once popular Romagnola sire born in 1969, between four to eight generations on both, the paternal and maternal side (Figure S1). Therefore, we applied an identical by descent mapping approach to fine-map the region containing the paunch calf syndrome mutation. Based on the pedigree records we hypothesized that the affected calves most likely were inbred to one single founder animal (Figure S1). Under this scenario the affected individuals were expected to be identical by descent (IBD) for the causative mutation and flanking chromosomal segments. To identify IBD regions between paunch calf syndrome cases we analyzed the cases for extended regions of homozygosity with simultaneous allele sharing. Only the disease-associated genome region on BTA 17 fulfilled our search criteria. All 16 cases were homozygous for the same disease-associated haplotype and shared identical alleles over 341 SNP markers corresponding to a 1.23 Mb interval (55,880,892–57,114,451 bp) on BTA 17 (Figure 2C). This cattle chromosome region contains 24 genes and loci according to the annotation of the corresponding human chromosome (HSA) 5 intervals (Figure 2D).

### A Missense Mutation in the KDM2B Gene is the Most Likely Causative Mutation

**Figure 3:**
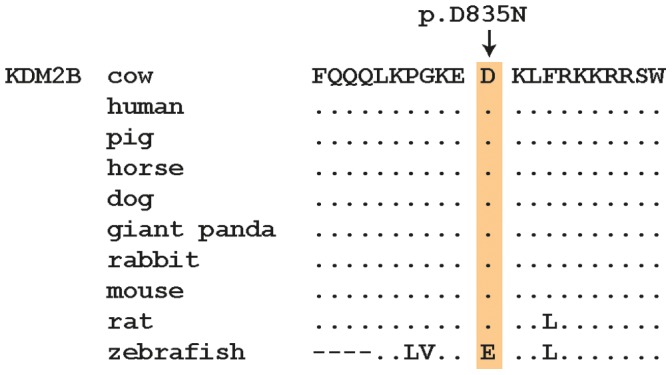
Multispecies alignment of the region around the KDM2B:p.D835N mutation.

To identify the mutation associated with paunch calf syndrome, we used a targeted sequence capture approach to re-sequence the critical 1.23 Mb interval in one affected calf. Sequencing coverage was limited to approximately 55% of the target region, as repetitive elements were masked during target enrichment probe design. Alignment of sequence reads showed that we obtained 98.6% of the single-copy sequences of the targeted BTA 17 interval, with 95.1% of the region covered by at least 50 high-quality reads. We mapped 17,986,641 sequences to the target region and a total of 8,030,795 sequence reads (44.6%) were reliably mapped to the target region with a mapping quality >20 resulting in a mean coverage of 406 for the probe design region. In total, 95% (87.4% with mapping quality >20) of the coding bases of the exome defined by UMD3.1 cattle genome annotation [8] were represented by at least 50 reads per base. Within the critical interval, we identified 400 homozygous sequence variants (382 SNPs and 18 indels) in comparison to the reference cattle genome sequence (Table S1). Visual inspection of the read-pairs revealed no indication for structural variants spanning coding regions. To distinguish potentially pathogenic mutations from other variants, we focused only on homozygous variants located in coding and splice site sequences, anticipating that variants in non-coding sequences were less likely to be pathogenic. Applying this filter, we identified two non-synonymous coding variants that were associated with paunch calf syndrome. These were a 12 bp deletion (c.704–716del) in the *ring finger protein 34, E3 ubiquitin protein ligase* gene (*RNF34*) leading to a truncated stretch of aspartic acid residues, and a SNP (c.2503G>A) in the *lysine (K)-specific demethylase 2B* gene (*KDM2B*) causing an aspartic acid to asparagine substitution (p.D835N) (Figure S2). The c.2503G>A transition is located in *KDM2B* exon 18 and the encoded residue is located between the CXXC zinc finger and F-box domains of the KDM2B protein in a region of very high sequence conservation (Figure 3).

**Figure 4:**
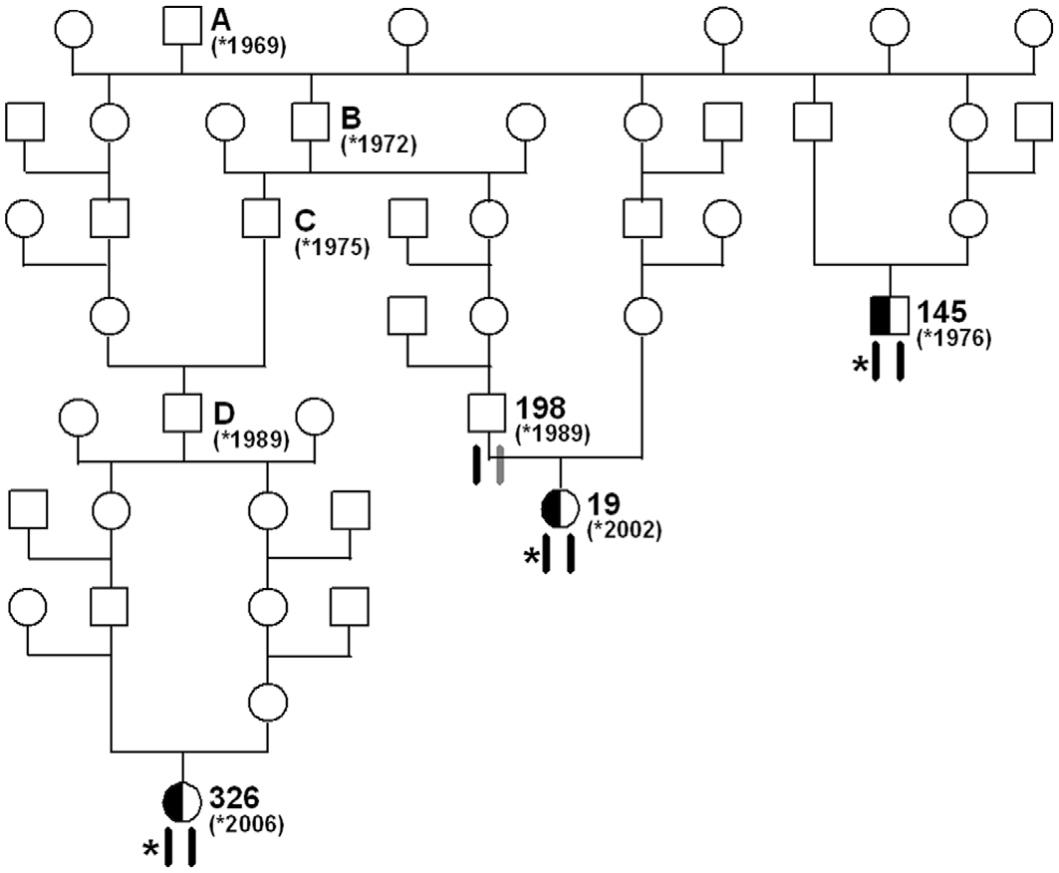
Pedigree of selected inbred Romagnola cattle. The special structure of livestock families facilitates the mutation identification: The sire A, born 1969, is the assumed founder animal for paunch calf syndrome. The paunch calf syndrome mutation (indicated by an asterisk) most likely occurred in the germline of sire A. Beneath the animals the two copies of BTA 17 are indicated. Black symbols represent the ancestral chromosome, on which the mutation occurred. Gray symbols represent any other wild-type copy of BTA 17. Due to inbreeding loops three animals (cow 326, cow 19, bull 145) have inherited two copies of the same chromosome from their common ancestor (sire A). These three animals were genotyped as heterozygous carriers of the *KDM2B* mutation. For the cow 19 it is shown that her paternal copy of the critical 1.23 Mb interval is still in its ancestral wild-type state as her father (bull 198) was tested *KDM2B* clear, while her maternal copy carries the paunch calf syndrome mutation. The BTA 17 haplotypes were confirmed by SNP genotyping of available samples from cow 326, cow 19, bull 198 and bull 145. Based on pedigree and marker data these three animals were identical-by-descent for the critical segment on BTA 17, with the only exception of the causative paunch calf syndrome mutation.

Both polymorphisms were further investigated by genotyping additional cases, parents and unrelated controls using Sanger sequencing (Figure S1, Figure S2). We found perfect concordance between the presence of the *KDM2B* c.2503G>A variant and the paunch calf syndrome phenotype (Table 1). Only one reported paunch calf syndrome carrier yielded a discordant genotyping result and was homozygous for the wildtype allele, which is most likely due to a phenotyping error of the offspring (Table 1). The *RNF34* deletion was not perfectly associated with paunch calf syndrome was thus excluded (Table 1). A total of 3 out of 255 Romagnola controls were genotyped as homozygous mutant for the *RNF34* deletion and should by implication be affected if that were the causative mutation, but they were only heterozygous for the *KDM2B* SNP. Interestingly, these 3 animals were inbred to the assumed paunch calf syndrome founder (Figure 4). We performed SNP array genotyping of these individuals and detected that they were homozygous for all tested markers in the critical interval on BTA 17. In other words their SNP array genotypes in the critical region were identical to the SNP array genotypes of paunch calf syndrome cases.

**Table 1:** Genotyping results for *RNF34* and *KDM2B* mutations.

		*RNF34* genotype (c.704–716del)		
		del/del	del/wt	wt/wt
Cases	(n = 65)	65		
Parents^a^	(n = 57)		56	1^b^
Romagnola controls	(n = 466)	3	172	291
Other breed controls	(n = 288)			288
		***KDM2B*** ** genotype (c.2503G>A)**		
		**A/A**	**AG**	**GG**
Cases	(n = 65)	65		
Parents^a^	(n = 57)		56	1^b^
Romagnola controls	(n = 466)		150	316
Other breed controls	(n = 288)			288

a Cattle recorded as parent of affected calves.

b For this cow only one single suspicious offspring was recorded by the breeding organization and the diagnosis of this calf had not been confirmed by necropsy. Genotyping of additional flanking markers revealed that this animal did not carry the disease-associated haplotype. Therefore, the reported calf most likely represented a phenocopy and the parent is indeed free of the deleterious mutation.

None of 523 healthy Romagnola cattle had the homozygous *KDM2B A/A* genotype (Table 1). The *KDM2B* variant was absent in all tested non-Romagnola cattle. It had an allelic frequency of 16% amongst 466 healthy Romagnola individuals that were not related to cases at the parent level. We carefully checked the available pedigree records of genotyped carriers and in all *A/G* animals we were able to identify at least one line of relationship to the assumed founder. The two oldest genotyped carriers were a grandson and a great-grandson of this sire, respectively (Figure S1).

## Discussion

Using a genome-wide association study approach followed by targeted re-sequencing, we have identified the *KDM2B* c.2503G>A variant as most likely causative mutation for the lethal multi-organ developmental dysplasia in homozygous mutant animals. We exploited the special structure of cattle families to identify the causative mutation by a purely genetic approach. The rich pedigree records in cattle breeding allowed us to identify the possible founder animal of paunch calf syndrome, a Romagnola bull born in 1969. A few generations later several cattle received two copies of the same chromosome segment from this bull due to inbreeding. We hypothesize that this sire was a germline mosaic as he transmitted copies of the BTA 17 chromosome which acquired the causative mutation, while the other copies stayed in the ancestral state (Figure S1; Figure 4). Three inbred animals, which were IBD for all tested markers across the critical interval, were heterozygous at the *KDM2B* mutation. The recognition of these animals was a key element in our discovery and illustrates once again the potential of livestock specific population structures for genetic research [6]. In addition, we detected mutation carriers born two or three generation after the assumed founder, which provide additional support for the hypothesis of the origin of the causative mutation. The DNA-based test for the detection of paunch calf syndrome carriers test been already adopted by the Italian Romagnola Breeders’ Association and all the top sires indexed in the annual catalogues are identified as to whether they are carriers of the paunch calf syndrome causative mutation. Since summer 2011, there has been a requirement for young bulls short-listed for entrance into the performance testing program to be tested for the mutation; carriers are excluded from the programme. The 32% carrier frequency within the examined Romagnola population is very high compared to other recessive traits we studied [6+7]. This might explain the recently observed outbreak of the paunch calf syndrome disease due to intensive usage of a limited number of artificial insemination sires and subsequent increase of inbreeding level.

Due to the recessive inheritance and the lethal effect of the mutation we hypothesized that most likely a loss of function mutation affecting the coding sequence of a gene would be responsible for paunch calf syndrome. Therefore, we subsequently concentrated on homozygous variants that were located within the coding sequences or within the splice sites of the annotated genes in the targeted region of the bovine genome. Our genetic data implicates *KDM2B* as a cause of paunch calf syndrome. Only the identified missense mutation in *KDM2B* co-segregates with the disease phenotype, and the re-sequencing of the entire paunch calf syndrome locus failed to identify other perfectly associated non-synonymous variants in transcribed sequences. The second non-synonymous variant in *RNF34* is most likely functionally neutral as we detected the homozygous mutant *RNF34* genotype in three normal control cattle. Unfortunately, there is no experimental crystal structure available to predict the structures of wildtype and mutant KDM2B proteins. The exchange of an acidic residue to an uncharged amino acid with the loss of a negative charge may disrupt the normal structure. Another possible structural effect could be that an aspartic acid residue accepts two hydrogen bonds instead of only one in case of an asparagine. In regard to the close vicinity of the mutant residue to the zinc finger domain of KDM2B the mutation could also affect its role as metal ligand.

The *KDM2B* gene (also known as *JHDM1B* and FBXL10) encodes a histone H3 lysine 36 dimethyl (H3K36me2)-specific demethylase [9], [10]. Histone methylation is one important transcription regulatory system that affects mammalian development and cell differentiation [11]. During the embryonic and fetal differentiation, chromatin modifications are important to drive the correct temporal expression of specific genes. Transcription factors act in concert with histone acetylatases and methylases to influence gene transcription by modifying the architecture of chromatin at specific loci [12]. Histone methylation had been thought of as an irreversible epigenetic mark until the first lysine specific histone demethylase LSD1 (also known as KDM1) was discovered [13]. Subsequent to the discovery of LSD1, another larger family of more than 30 histone demethylases sharing a motif designated the Jumonji C (JmjC) domain, structurally different from LSD1 but including KDM2B, was described [10]. This enzyme catalyzes the removal of methyl groups from a specific lysine residue on histone H3 and is capable of suppressing cellular senescence [9]. Recently, it was shown that KDM2B acts in concert with other key reprogramming factors in early gene activation and promotes induced pluripotent stem cell generation from somatic cells [14]. To our knowledge, no human patients with *KDM2B* mutations have so far been identified. Several other JmjC-domain-containing histone demethylases have been implicated in disease including prostate cancer [15] and X-linked intellectual disability [16+17]. *Kdm2b*-deficient mice exhibit failure of neural tube closure, resulting in exencephaly and death shortly after birth [18]. These recent findings in mice suggest that the *KDM2B* gene is involved in neural development. Our report indicates that *KDM2B* function is also important for the development of endo- and mesodermal derived organs like bone and liver.

In conclusion, our results suggest that the *KDM2B* c.2503G>A variant represents the causative mutation for paunch calf syndrome in Romagnola cattle. Especially, the genetic makeup of three inbred cattle provides extremely strong support for the causality of this mutation. Selection against this mutation can now be used to eliminate paunch calf syndrome from Romagnola cattle in production systems. Furthermore, we discovered a hint for a possible essential role of this histone demethylase during the development of endo- and mesodermal organs. This study highlights the potential of naturally-occurring mutants in domestic animals to gain further insights into gene functions.

## Materials and Methods

### Ethics Statement

All animal work has been conducted according to the national and international guidelines for animal welfare. There is no permit number as this study is not based on an invasive animal experiment. All cattle owners agreed that the samples can be used for our study. The data were obtained during diagnostic procedures that would have been carried out anyway. This is a very special situation in veterinary medicine. As the data are from client-owned cattle that underwent veterinary exams, there was no “animal experiment” according to the legal definitions in Italy.

### Animals

We collected samples from 65 paunch calf syndrome affected calves (35 male, 30 female) from different farms. For most of the cases detailed pedigree records were available and their relationship is illustrated in Figure S1. The phenotype classification was primarily based on the owners’ reports. Most of the affected animals were seen by a veterinarian and for some cases necropsy results were available. Additionally, we collected 57 samples recorded as parents (14 sires, 43 dams) of affected offspring and 466 healthy Romagnola cattle (352 male, 114 female) resulting in a total of 588 samples from this breed. For the association study we also used 288 normal cattle from 25 genetically diverse *Bos taurus* breeds.

### Genome Wide Association Mapping

Genotyping was performed using the BovineHD BeadChip (Illumina), including 777,962 evenly distributed SNPs on the 29 bovine autosomes and X chromosome (median distance between markers: 2.68 kb), and standard protocols recommended by the manufacturer. A total of 241,791 SNPs were excluded based on genotyping rate (call frequency <80%; 5,949 SNPs) and minor allele frequency (MAF <10%; 237,307 SNPs). For single-marker association studies the genotype frequencies at 536,171 SNPs were compared between cases and controls using a standard allelic association test implemented with PLINK [19]. Genome-wide corrected empirical p-values were determined applying the max(T) permutation procedure implemented in PLINK with 500,000 permutations. To identify extended homozygous regions with allele sharing across all affected animals the options –homozyg-group and –homozyg-match were applied. All given positions correspond to the UMD3.1 cattle genome assembly [8]. The corresponding human chromosome segments (build 37.2) were identified by BLASTN searches of bovine SNP flanking sequences to the human genome sequence.

### Sequence Capture Enrichment

DNA fragmentation was conducted using a microTube in a Covaris S2 instrument, resulting in 100 to 150 bp average fragment length at the sample peak, as monitored using an Agilent Bioanalyzer. Ends were repaired using a DNA end polishing enzyme mixture (Agilent). Double stranded custom adaptors containing barcode sequences (sequences available upon request; Agilent) were ligated to the blunt ended genomic DNA fragments using T4 DNA ligase (Agilent). Resulting fragments were purified by elution from E-gel (Invitrogen) to enrich approximately 200 bp length fragments. These fragments were nick translated and amplified by PCR using primers specific for adaptor sequences for 12 cycles using Platinium PCR SuperMix High Fidelity (Invitrogen) standard PCR conditions.

PCR products were then enriched using a custom SureSelect 120 bp probe set in solution (Agilent). Probes were designed using eArray web-based software (Agilent) to cover a 1.74 Mb region from 55,853,187 to 57,599,455 bp which includes the homozygosity region of BTA 17 and three additional segments of the bovine genome with a total size of 2.46 Mb as control. Repetitive elements were excluded from the probe design. A total of 56,923 probes were created for the target regions. For hybridization, we followed the manufacturer’s directions with the following modifications. The library was hybridized at 65°C for 24 h, and then purified using streptavidin M280 beads (Dynal, Invitrogen). Eluted DNA was amplified using adaptor-specific primers for 12 cycles as before hybridization. Enriched products were analyzed using an Agilent Bioanalyzer to determine their size and concentration.

### High Throughput Sequencing, Read Mapping and Variant Calling

The paired-end library was sequenced on each end for 50 and 35 cycles, respectively, on one quarter of a slide on a SOLiD4 sequencer (Life Technologies). We collected 383 million reads (191.5 million pairs) and aligned them to the bovine genome (UMD3.1) using BioScope (Life Technologies) with default settings. Alignment data were stored in BAM format and visualized using IGV software [20]. Duplicate pairs were removed, and only reads with reliable alignment quality >20 were used for the variant (SNP and small indel) detection. Sequence variants were detected using BioScope software. High-confidence variants were supported by 8 or more high-quality aligned reads on both strands. Lower confidence variants for low coverage regions were required to be supported by at least 4 reads on each strand.

### Sanger Re-sequencing

Two variants were genotyped by re-sequencing of targeted PCR products using Sanger sequencing technology. PCR products were amplified using AmpliTaqGold360Mastermix (Life Technologies). PCR products were directly sequenced on an ABI 3730 capillary sequencer (Life Technologies) after treatment with exonuclease I and shrimp alkaline phosphatase. Sequence data were analyzed with Sequencher 4.9 (GeneCodes).

## Supporting Information

Figure S1 **Pedigree tree of 46 paunch calf syndrome cases with available family records.** Filled symbols represent paunch calf syndrome affected calves, open symbols represent normal cattle. DNA samples were available only for the numbered individuals. Animals genotyped as carriers of the *KDM2B* c.2503G>A mutation are shown with half-filled symbols. The mutation probably occurred in sire A and was spread into the population by his son B, grandson C, and great-grandson D. Most of the mothers of affected calves are related to these bulls.
(EPS)

Figure S2 ***KDM2B*** **mutation.** (A) Visualization in the IGV browser: Individual reads overlapping with the mutation are displayed. Seventy of eighty-one reads show the homozygous mutation at genomic position BTA 17∶56′010′031. (B) Sanger sequencing electropherograms of a wildtype control animal, a heterozygous carrier and a PCS affected calf are shown. The protein translation of the wild-type and mutant sequence is shown above the electropherograms.
(PDF)

Table S1 **Sequence variants in comparison to the reference cattle genome sequence.**
(XLSX)
