# Supplementary figures and images for: KDM2B Is Implicated in Bovine Lethal Multi-Organic Developmental Dysplasia
Source: PLoS One. 2012 Sep 27;7(9):e45634. doi: 10.1371/journal.pone.0045634 (PMC3459949; doi:10.1371/journal.pone.0045634)

**A**

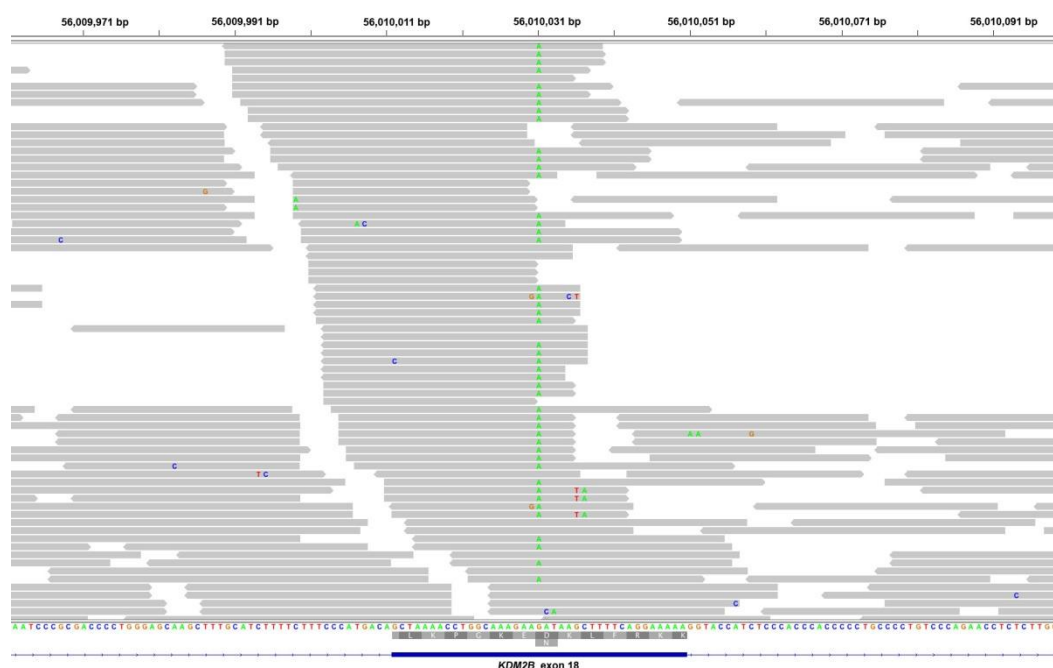

**B**

**homozygous wildtype**

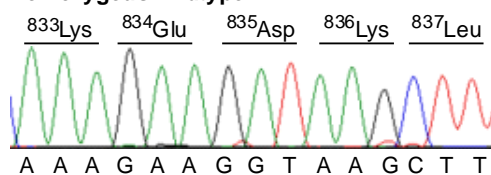

**heterozygous carrier**

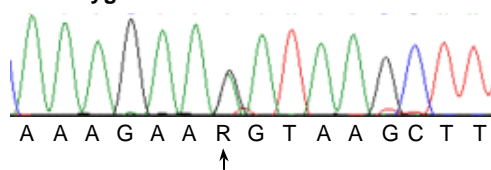

**homozygous mutant**

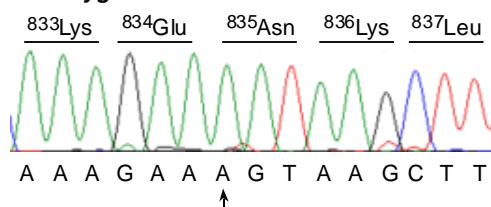

Supplement: Figure S2 — KDM2B mutation. (A) Visualization in the IGV browser: Individual reads overlapping with the mutation are displayed. Seventy of eighty-one reads show the homozygous mutation at genomic position BTA 17∶56′010′031. (B) Sanger sequencing electropherograms of a wildtype control animal, a heterozygous carrier and a PCS affected calf are shown. The protein translation of the wild-type and mutant sequence is shown above the electropherograms. (PDF) [file pone.0045634.s002.pdf]
